# Transcriptomic analysis reveals novel mechanisms of SARS‐CoV‐2 infection in human lung cells

**DOI:** 10.1002/iid3.366

**Published:** 2020-10-30

**Authors:** Shaomin Yang, Songbin Wu, Zhijian Yu, Jiabin Huang, Xia Zhong, Xiaodong Liu, Hua Zhu, Lizu Xiao, Qiwen Deng, Wuping Sun

**Affiliations:** ^1^ Department of Pain Medicine and Shenzhen Municipal Key Laboratory for Pain Medicine, Shenzhen Nanshan People's Hospital The 6th Affiliated Hospital of Shenzhen University Health Science Center Shenzhen China; ^2^ Jinan University Guangzhou China; ^3^ Department of Infectious Diseases and Shenzhen Municipal Key Laboratory for Endogenous Infection, Shenzhen Nanshan People's Hospital The 6th Affiliated Hospital of Shenzhen University Health Science Center Shenzhen China; ^4^ Department of Anaesthesia and Intensive Care The Chinese University of Hong Kong Hong Kong; ^5^ Rutgers New Jersey Medical School Newark New Jersey USA

**Keywords:** ACE2, coronavirus, COVID‐19, interferon response, pyrimidine metabolism, SARS‐COV‐2, steroid biosynthesis

## Abstract

**Background:**

Severe acute respiratory syndrome coronavirus clade 2 (SARS‐CoV‐2) is a single‐stranded RNA virus responsible for the global pandemic of the coronavirus disease‐2019 (COVID‐19). To date, there are still no effective approaches for the prevention and treatment of COVID‐19.

**Objective:**

The present study aims to explore the possible mechanisms of SARS‐CoV‐2 infection in human lung cells.

**Methods:**

Data interpretation was conducted by recruiting bioinformatics analysis, including Gene Ontology and Kyoto Encyclopedia of Genes and Genomes pathways analysis using downloaded data from the NCBI Gene Expression Omnibus database.

**Results:**

The present study demonstrated that SARS‐CoV‐2 infection induces the upregulation of 14 interferon‐stimulated genes, indicative of immune, and interferon responses to the virus. Notably, genes for pyrimidine metabolism and steroid hormone biosynthesis are selectively enriched in human lung cells after SARS‐CoV‐2 infection, suggesting that altered pyrimidine metabolism and steroid biosynthesis are remarkable, and perhaps druggable features after SARS‐CoV‐2 infection. Besides, there is a strong positive correlation between viral ORF1ab, ORF6, and angiotensin‐converting enzyme 2 (ACE2) expression in human lung cells, implying that ACE2 facilitates SARS‐CoV‐2 infection and replication in host cells probably through the induction of ORF1ab and ORF6.

## Graphical abstract

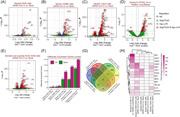

## 1 INTRODUCTION

The prevalence of coronavirus disease‐2019 (COVID‐19), which is caused by severe acute respiratory syndrome coronavirus clade 2 (SARS‐CoV‐2), at the end of 2019 became a global pandemic.^1^ According to the World Health Organization (WHO) data, the latest infection cases in the world have reached more than 37.4 million, with the reported deaths of more than 1,075,000 individuals.^2^ Until now, the prevalence of COVID‐19 seems still to be increasing exponentially. Therefore, the effective approaches for the prevention and treatment of COVID‐19 are urgently needed.

Coronaviruses are a family of enveloped, positive‐stranded RNA viruses, which can infect various vertebrate hosts, including bats, dogs, and humans.^3^ Human beings have suffered and experienced three crises caused by coronaviruses in the new millennium. The first is SARS‐CoV‐1, which has caused a pandemic in 2003.^4^ The second is named Middle East respiratory syndrome‐CoV, the Middle East respiratory syndrome‐related coronavirus.^4^ SARS‐CoV‐2 has been found at the end of 2019, and its pathogenicity is even higher than the first two coronaviruses.^5^ SARS‐CoV‐2 causes the ongoing COVID‐19 pandemic. The threat of SARS‐CoV‐2 is due to the impact of the human respiratory system.^6^ The common syndromes of COVID‐19 in humans are usually fever, cough, and dyspnea.^7^ Infection with SARS‐CoV‐2 also causes acute respiratory distress syndrome and acute lung injury, which are the major factors that result in a malfunction of lung and death in elder and weak people.^8^ To date, human beings are suffering from the COVID‐19 crisis, but there are still no effective approaches for the prevention and treatment of COVID‐19 due to a lack of knowledge of SARS‐CoV‐2 pathogenesis in the infected cases. Therefore, there is a pressing urgency to understand the pathogenesis of COVID‐19, exploration of the characters and signatures on the molecular level, subcellular level, cellular function, and biopsy network activities of SARS‐CoV‐2 infection.

To date, several papers have already reported the transcriptional changes in the samples with SARS‐CoV‐2 infection from in vitro, ex vivo, and in vivo systems.^9^, ^10^ However, these papers did not fully interpret the transcriptional signatures on the molecular level, subcellular level, cellular function, and network activities. Moreover, angiotensin‐converting enzyme 2 (ACE2) has already been reported to be a functional receptor, which is critical for SARS‐CoV entry into host cells.^11^, ^12^ ACE2 is known to be expressed on nonimmune cells, such as respiratory and intestinal epithelial cells, endothelial cells, kidney cells (renal tubules), and cerebral neurons.^13^, ^14^, ^15^ In the present study, we performed data interpretation by recruiting bioinformatics analysis, including Gene Ontology (GO) and Kyoto Encyclopedia of Genes and Genomes (KEGG) pathways analysis using downloaded data from the NCBI Gene Expression Omnibus (GEO) database, as a recent paper reported.^16^ Furthermore, we compared the viral infection and replication efficacy on different types of human lung cells with or without modification of ACE2 expression.

## 2 MATERIALS AND METHODS

### 2.1 Transcriptome analysis

The datasets RNA consisting of sequencing data of four human lung‐derived cell lines, that is, A549, normal human bronchial epithelial (NHBE), Calu3, and ACE2 overexpressing A549 (with or without SARA‐CoV‐2 injection, three repeats), and four human lung biopsies (two healthy subjects and two COVID‐19 patients) were downloaded from the NCBI GEO database (accession number GSE147507), which was submitted by Blanco‐Melo et al. recently.^16^

The quality of the FASTQ files generated from the libraries was checked using Fast QC (http://www.bioinformatics.babraham.ac.uk/projects/fastqc) and summary statistics reporting by MultiQC v1.7 (https://multiqc.info/). The RNA‐seq workflows were running on two Intel W‐3175X CPUs with 128 GB of memory.^17^ After removing low‐quality, adapter‐polluted, and high content of unknown base (N) read, the clean reads data were aligned to the SARS‐CoV‐2 genome (NC_045512.2) using Bwa Aligner.^18^ The counts of mapped reads for each gene were calculated using FeatureCounts^19^ (http://bioinf.wehi.edu.au/featureCounts/) from the SAM files. The differentially expressed genes (DEGs) were defined as genes with at least twofold changes between groups and an adjusted *p* value less than .05 using DESeq2.^20^ ClusterProfiler,^21^ which uses a modified Fisher's exact test followed by Benjamini–Hochberg multiple hypotheses testing corrections and corrected *p* value cutoff of 0.05, was used to perform gene functional annotation clustering human genes as background, and default options and annotation categories. Significantly enriched KEGG pathways were identified using a hypergeometric test and Benjamini–Hochberg FDR correction.^22^, ^23^

### 2.2 Statistical analysis

An empirical Bayesian analysis was performed to shrink the dispersions toward a consensus value, effectively borrowing information between genes.^24^, ^25^ Genes with a *q* value lower than .05 and a fold change greater than 2 were considered differentially expressed. Group data are presented as the mean ± *SEM*. Pearson's product‐moment correlation analysis was conducted to evaluate the statistical differences. Results were considered significant at *a p* value of less than .05.

## 3 RESULTS

### 3.1 Dysregulated transcripts occurred in SARS‐CoV‐2‐infected human lung cell lines and human lung tissues

We first conducted quality control of the sequence data downloaded from the GEO database. We counted the number of identified expressed genes and calculated its proportion and distribution to each sample's total gene number as Figure S1A. The principal component analysis was performed to assess the variation of datasets. Samples with or without SARS‐CoV‐2 injection were separated (Figure S1B). Besides, we calculated the correlation value between every two samples based on normalized expression results and drew a correlation heatmap (Figure S1C). These results suggest that the sequence data downloaded from the GEO database can be used for subsequent analysis.

**Figure 1:**
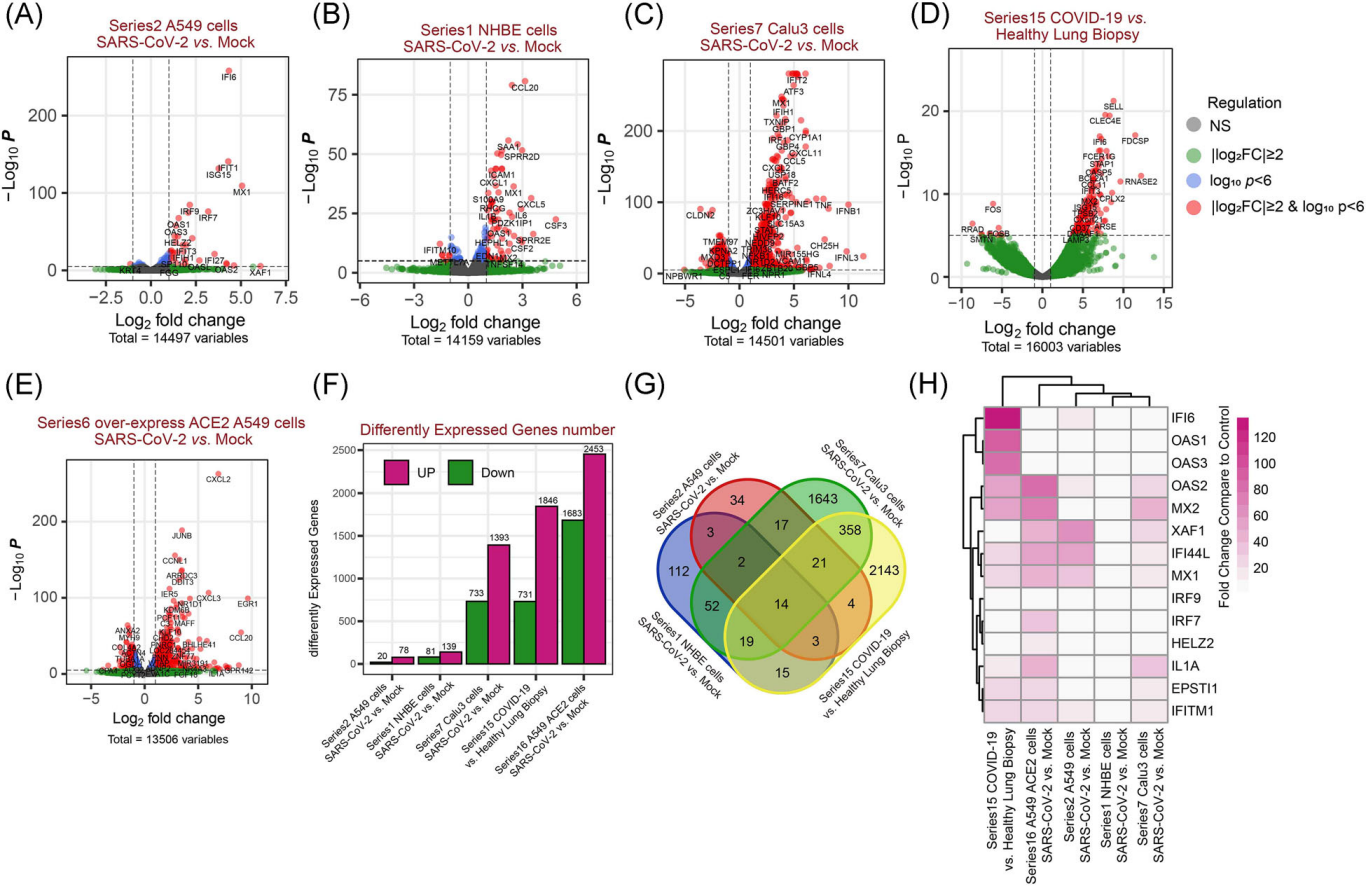
Many differentially expressed genes in a variety of types of human lung cells. (A–E) Volcano plot for the cells with mRNA expression differences in each paired group. Log_2_ (fold change) is plotted as the abscissa, and log_10_ (*p* value) is plotted as the ordinate. Differentially expressed genes (DEGs) are indicated in red. (F) The total up‐ or downregulated DEGs in each paired group. (G) A Venn diagram presents the number of unique or shared DEGs in each paired group. (H) A fold‐change heatmap of the shared 14 genes from each human lung cells with SARS‐CoV‐2 infection. mRNA, messenger RNA; SARS‐CoV‐2, severe acute respiratory syndrome coronavirus clade 2

DEGs were identified from the same cell lines (mock vs. SARS‐CoV‐2 infection) or human biopsies (healthy vs. COVID‐19) and used for functional annotation. All DEGs were demonstrated in volcano plots (Figure 1A–E). In A549 cells (human lung type II alveolar epithelial‐like cells), there were 78 upregulated DEGs and 20 downregulated DEGs (Figure 1A). A total of 139 genes were significantly upregulated, while 81 genes were downregulated in NHBE cells after viral infection (Figure 1B). DEGs in Calu‐3 (human adenocarcinoma lung epithelial) cells were shown in Figure 1C, with 1,393 genes upregulated and 733 genes downregulated. When COVID‐19 patients compared with non‐COVID‐19 patients, there were 1,846 upregulated DEGs and downregulated 731 DEGs (Figure 1D). We have also analyzed the DEGs between ACE2 overexpressing A549 cells with or without SARS‐CoV‐2 infection (Figure 1E). Interestingly, these results revealed that the number of up‐ or downregulated DEGs are dramatically increased compared with A549 cells without ACE2 overexpression. A Venn diagram was plotted to identify the common or unique DEGs in human lung cells and lung biopsies (Figure 1G). A total of 14 common DEGs were identified. Notably, most of them are interferon‐stimulated genes (ISGs). The heatmap demonstrating the expression levels of 14 common DEGs is plotted in Figure 1H. All these 14 genes were upregulated in human lung cells and tissues after SARS‐CoV‐2 infection.

### 3.2 GO analysis reveals that immune and interferon responses were involved by SARS‐CoV‐2 infection

**Figure 2:**
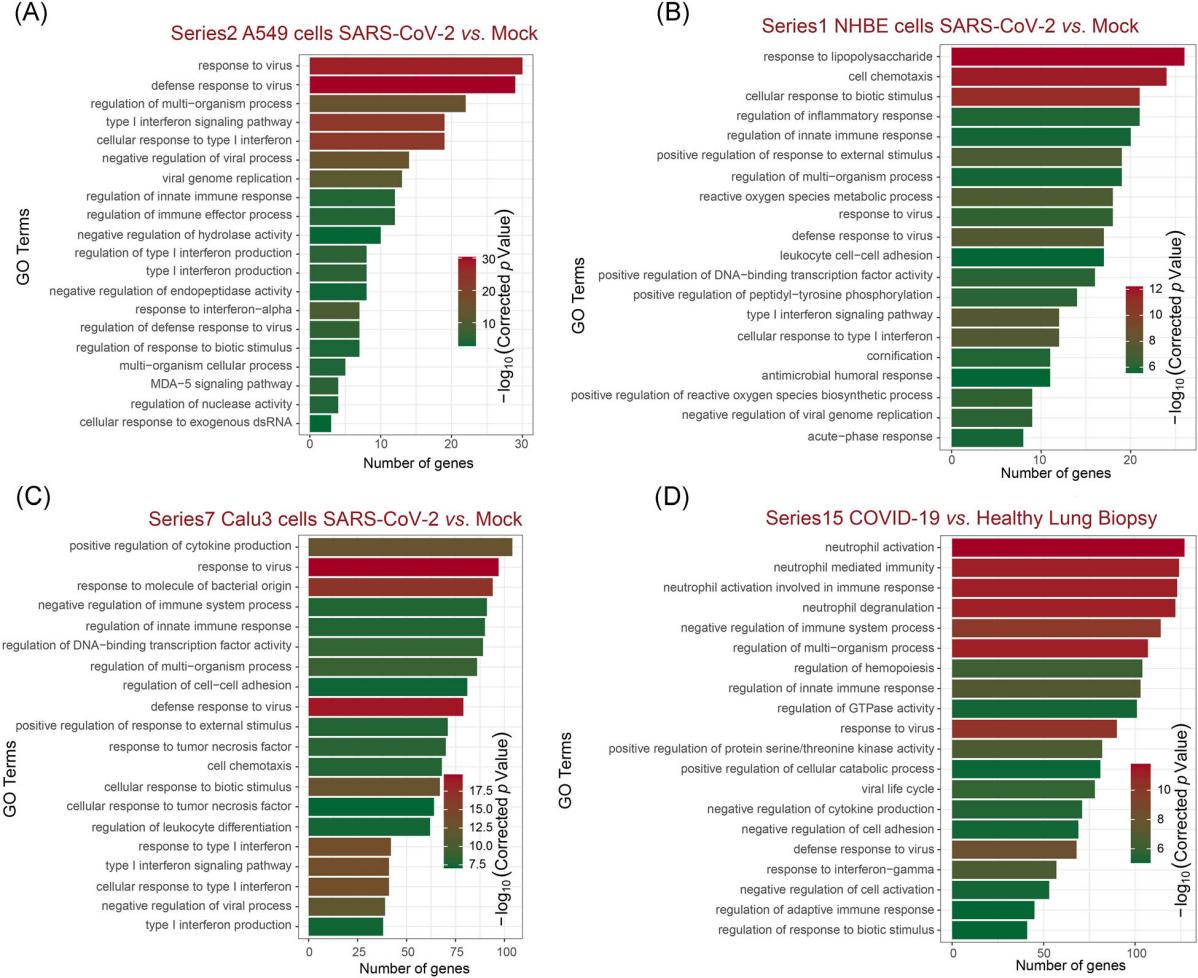
Functional analyses of DEGs by Gene Ontology classifications. The top 20 significantly enriched Gene Ontology (GO) terms in biological process enrichment of A549 cells (A), NHBE cells (B), Calu3 cells (C), and COVID‐19 lung biopsy (D) after SARS‐CoV‐2 infection. The enriched gene number as the abscissa and GO terms is plotted as the ordinate. DEG, differentially expressed gene; NHBE, normal human bronchial epithelial; SARS‐CoV‐2, severe acute respiratory syndrome coronavirus clade 2

GO analysis was carried out with DEGs to delineate the potential SARS‐CoV‐2 pathogenesis (Figure 2). In both human lung cell lines (Figure 2A–C) and human lung biopsies (Figure 2D), the biological processes (BP) are mainly associated with “immune response,” “defense response to the virus,” and “interferon response,” highlighting that innate immune responses to the virus are the common events after SARS‐CoV‐2 infection (Figure 2). As these common BP are highly enriched in cell lines of nonimmune cells, this result implied that lung‐derived cells (e.g., lung epithelial cells) are partially contributing to the harmful immune responses during the pathogenesis of COVID‐19. Meanwhile, it should be noted that a series of neutrophil related BP (such as neutrophil activation and neutrophil degranulation) were the most significantly associated BP in human tissues, inferring that immune cells, particularly neutrophils, might play a role in COVID‐19. Molecular function annotations revealed the enrichment of “receptor‐ligand activity,” “cytokine activity,” and “chemokine activity” in the list of DEGs, implying that cytokine and chemokines are the representative mediators of inflammatory responses in human lung cells with SARS‐CoV‐2 infection (Figure S2). However, for cellular component annotations, no common cellular components were identified among different sample pairs, suggesting various cellular components are involved in different conditions of human lung cells with SARS‐CoV‐2 infection (Figure S2).

### 3.3 Pyrimidine metabolism and steroid biosynthesis are involved in SARS‐CoV‐2 infection

**Figure 3:**
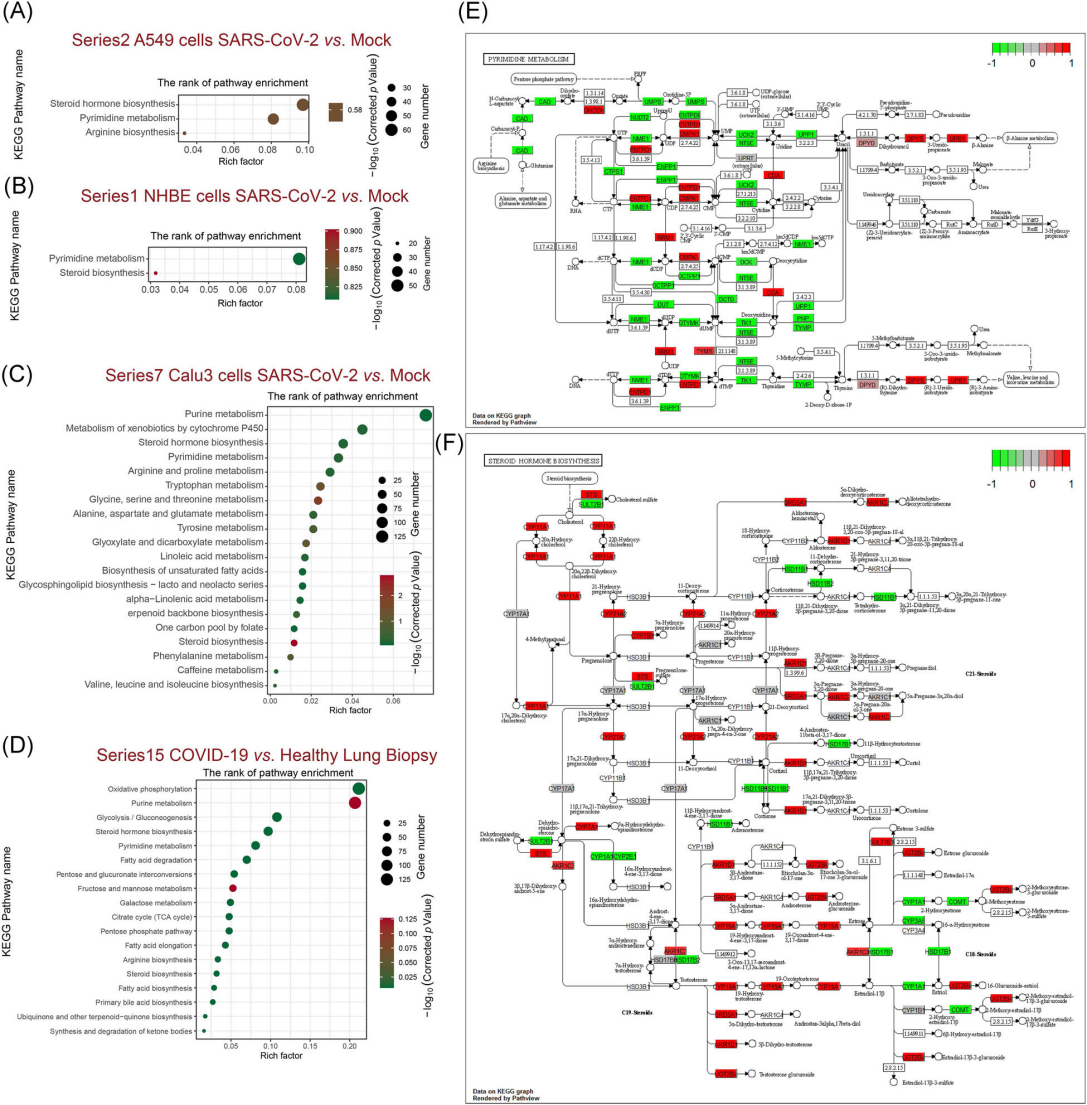
Kyoto Encyclopedia of Genes and Genomes (KEGG) classifications of DEGs in varies types of human lung cells. The comparison of pathway enrichment in A549 cells (A), NHBE cells (B), Calu3 cells (C), and COVID‐19 lung biopsy (D) after SARS‐CoV‐2 infection. It showed the top 20 significantly enriched KEGG pathways. The rich factor as the abscissa and KEGG terms is plotted as the ordinate. The functional analysis of the DEGs enriched in the pathways of pyrimidine metabolism (E) and steroid hormone biosynthesis (F) were shown. DEG, differentially expressed gene; NHBE, normal human bronchial epithelial; SARS‐CoV‐2, severe acute respiratory syndrome coronavirus clade 2

Next, we conducted KEGG pathway enrichment using ClusterProfiler. The differential genes, of particular interest, were significantly enriched for “pyrimidine metabolism,” “steroid biosynthesis,” and “steroid hormone biosynthesis,” which are common characters in lung‐derived cells or tissues infected by SARS‐CoV‐2, but not by the respiratory syncytial virus (RSV), influenza A virus (IAV), and human parainfluenza virus 3 (HPIV3; Figures 3A–D and S3A–C). These results suggested that altered pyrimidine metabolism and steroid biosynthesis are remarkable, and perhaps druggable features after SARS‐CoV‐2 infection. Besides, DEGs in Calu3 cells and human lung biopsies are also associated with multiple pathways involved in purine and amino acid metabolism. These results suggested that human lung cells may have occurred nucleotide, amino acid, lipid metabolism disorders upon SARS‐CoV‐2 infection. We further conducted a functional analysis of the DEGs enriched in the pathways of pyrimidine metabolism (Figure 3E) and steroid hormone biosynthesis (Figure 3F). The results showed that the DEGs enriched for the pyrimidine metabolism pathway mainly corresponded to an amino acid metabolism, including arginine biosynthesis, alanine metabolism, valine, leucine, and isoleucine metabolism. DEGs enriched in the steroid hormone biosynthesis pathway respond to progesterone, corticosterone/aldosterone, cortisol/cortisone, and estrone.

### 3.4 The infection efficacy of SARS‐CoV‐2 was correlated to ACE2 expression levels

**Figure 4:**
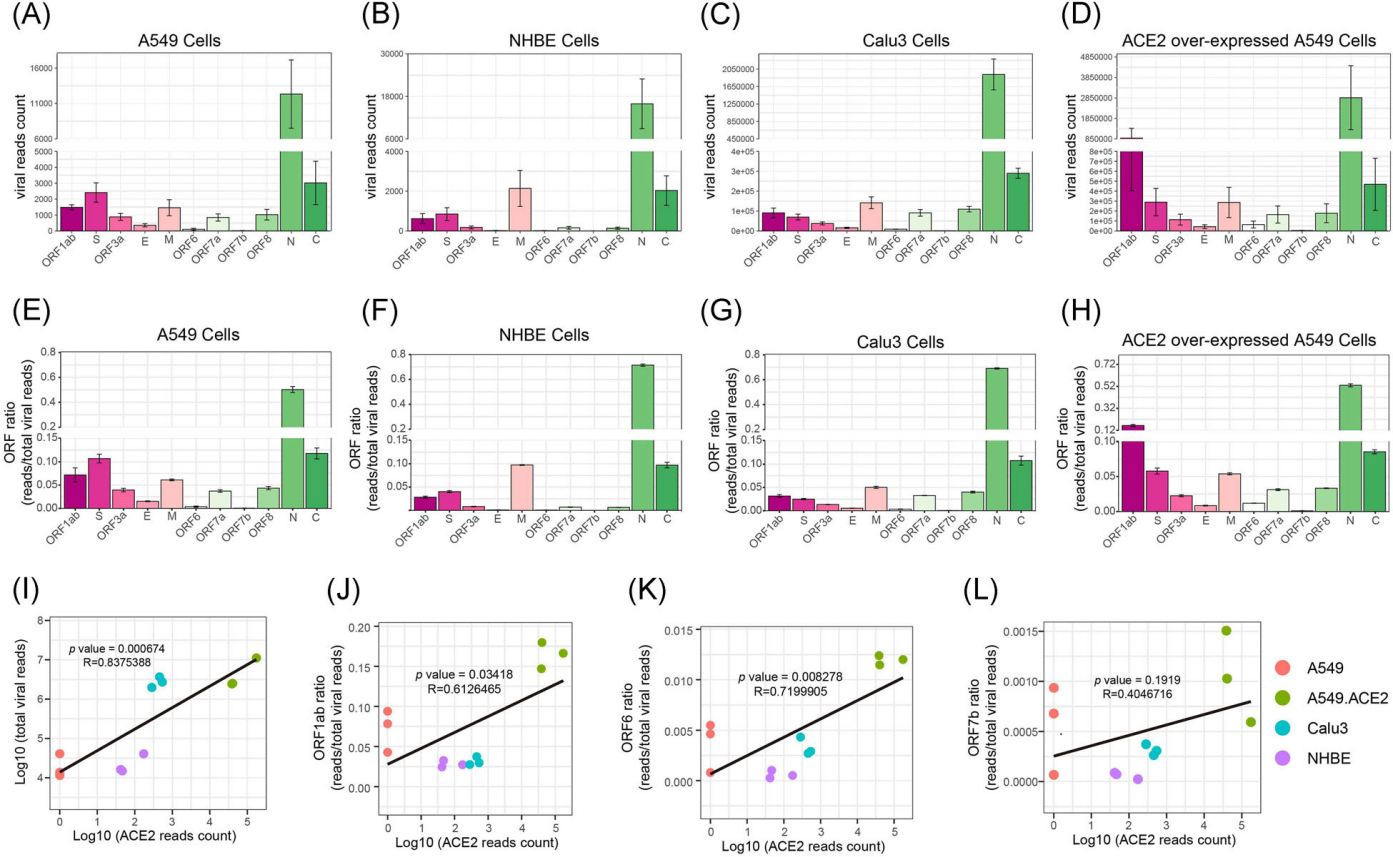
Quantitation of viral gene expression of SARS‐CoV‐2 in varies types of human lung cells after infection. The read‐count of each open read frame (ORF) in A549 cells (A), NHBE cells (B), Calu3 cells (C), and ACE2 over‐expressing A549 cells (D) after SARS‐CoV‐2 infection. The read‐count ratio of each ORF in A549 cells (E), NHBE cells (F), Calu3 cells (G), and ACE2 over‐expressing A549 cells (H) after SARS‐CoV‐2 infection. (I) The regression analysis of total viral read‐count and the expression of ACE2. The regression analysis of ORF1ab (J), ORF6 (K), and ORF7b (L) read‐count ratio and the expression of ACE2. Pearson's product‐moment correlation. ACE2, angiotensin‐converting enzyme 2; NHBE, normal human bronchial epithelial; SARS‐CoV‐2, severe acute respiratory syndrome coronavirus clade 2

It has been reported that ACE2 is the entry receptor that facilitates SARS‐CoV‐2 entry into target cells.^26^, ^27^ We explored the impact of ACE2 amounts in SARS‐CoV‐2 infection. We calculated the read‐count of each viral open read frame (ORF) after SARS‐CoV‐2 infection in A549 cells (Figure 4A), NHBE cells (Figure 4B), Calu3 cells (Figure 4C), and ACE2 overexpressing A549 cells (Figure 4D). The results demonstrated higher SARS‐CoV‐2 replication in NHBE cells, Calu3 cells than in A549 cells, although there is no expression of ACE2 in A549 cells according to the results of ACE2 read‐count and reverse transcription polymerase chain reaction (Figure 4SA,B). On the other hand, SARS‐CoV‐2 replication was dramatically increased in ACE2 over‐expressing A549 cells. Moreover, the ratio of each ORF reads in total virus reads was shown after SARS‐CoV‐2 infection in A549 cells (Figure 4E), NHBE cells (Figure 4F), Calu3 cells (Figure 4G), and ACE2 overexpressing A549 cells (Figure 4H). A regression analysis indicated a nearly linear correlation between the total virus reads and ACE2 expression in host cells (Figure 4I). Furthermore, compared to the ratio of each ORF in A549 cells, the ratio of ORF1ab and ORF6 looked increased in ACE2 overexpressing A549 cells. We examined the relationship between viral ORF1ab, ORF6, and ACE2 expression in human lung cells. The results indicated that there is a strong positive correlation between viral ORF1ab (Figure 4J), ORF6 (Figure 4K), and ACE2 expression in human lung cells, but not ORF7b (Figure 4L).

## 4 DISCUSSION

SARS‐CoV‐2 is a single‐stranded, positive‐sense RNA virus with a genome size of around ∼30 kb, responsible for the global pandemic of the COVID‐19 since the end of 2019.^28^, ^29^ SARS‐CoV‐2 poses an imminent threat to public health and the world's economy due to no approved therapeutic approaches or vaccines are available at present. However, lacking the pathogenesis of SARS‐CoV‐2 is problematic, given the urgent need for effective therapies against the emergence of COVID‐19. To understand the potential pathogenesis which SARS‐CoV‐2 caused, its transcriptomic architecture is warranted to be elucidated. In this study, we analyzed the transcriptomic data from various human lung cells and human lung biopsies with SARS‐CoV‐2 infection, downloaded from a recently published paper.^16^

DEG screening results indicated that many genes were altered in human lung cells and biopsies upon infection of SARS‐CoV‐2, suggesting the successful infection of SARS‐CoV‐2. Transcriptome changes result in the proteome alteration in cells, which subsequently affect the molecular and cellular functions. A Venn diagram showed that there are 14 DEGs shared in the infected human lung cells and biopsies. These 14 genes, including 2'‐5'‐oligoadenylate synthetase 1, interferon regulatory factor 7, XIAP associated factor 1, 2'‐5'‐oligoadenylate synthetase 2, interferon regulatory factor 9, helicase with zinc finger 2, interferon‐alpha inducible protein 6, interferon‐induced protein 44 like, interleukin 1 alpha, epithelial‐stromal interaction 1, MX dynamin‐like GTPase 1, the interferon‐induced transmembrane protein 1, 2'‐5'‐oligoadenylate synthetase 3, and MX dynamin‐like GTPase 2, are mainly ISGs, suggesting transcriptomic pattern changes are consistent in various types of human lung cells after SARS‐CoV‐2 infection.

GO analysis revealed that the innate immune responses induced by the virus are common in various human lung cells after SARS‐CoV‐2 infection. Meanwhile, neutrophil activation and neutrophil degranulation are the most significantly associated biological processes in human tissues, inferring that immune cells, particularly neutrophils, might play a role in COVID‐19. Molecular function annotations revealed that cytokine and chemokines are the representative mediators of inflammatory responses in human lung cells with SARS‐CoV‐2 infection. These results coincide with the previous reports that the induction of a cytokine storm is the root cause of pathogenic inflammation in COVID‐19.^30^, ^31^

Recent studies have revealed that pyrimidine metabolism participates in SARS‐CoV‐2 infection.^32^, ^33^ In the present study, KEGG analysis results demonstrated that pyrimidine metabolism and steroid hormone biosynthesis are the major pathways involved in the host cells after SARS‐CoV‐2 infection. These results suggested that altered pyrimidine metabolism and steroid biosynthesis are remarkable, and perhaps druggable features after SARS‐CoV‐2 infection. Besides, DEGs in Calu3 cells and human lung biopsies are also associated with multiple pathways involved in purine and amino acid metabolism. These results suggested that nucleotide, amino acid, lipid metabolism disorders may occur in human lung cells upon SARS‐CoV‐2 infection. The functional analysis uncovered that amino acid metabolisms are involved in pyrimidine metabolism. And DEGs enriched in the steroid hormone biosynthesis pathway respond to progesterone, corticosterone/aldosterone, cortisol/cortisone, and estrone. Another interesting finding is that nucleotide metabolism and hormone biosynthesis was triggered in human lung cells uniquely with SARS‐CoV‐2 infection, but not RSV, IAV, and HPIV3 infections, which could explain why that SARS‐CoV‐2 infection‐induced more lethality and severity symptoms in COVID‐19 patients, compared with common influenza. Our results also supported the clinical observations that COVID‐19 patients critically need nutritional intervention and mechanical ventilators, finally probably due to the caused metabolism disorders.^34^, ^35^ Therefore, more attention should be given to the threat of COVID‐19.

ACE2 is the entry receptor that facilitates SARS‐CoV‐2 entry into target cellsWelcome.^26^, ^27^ An interesting phenomenon we observed in the present study is that SARS‐CoV‐2 read‐count (infection and replication) in host cells is dramatically higher than in NHBE cells, Calu3 cells than in A549 cells. Another evidence is that SARS‐CoV‐2 replication was also dramatically increased in ACE2 over‐expressing A549 cells. Regression analysis results confirmed a strong positive correlation between the expression of ACE2 in host cells and SARS‐CoV‐2 replication. These results suggested that the high efficacy of ACE2, which facilitates the infection of SARS‐CoV‐2 in host cells. It has also been reported that ACE2 expression increases in varies types of human lung cells, including airway epithelial cells, alveolar AT2 cells, and submucosal gland secretory cells with increasing age, male gender, and smoking by a meta‐analysis of single‐cell RNA‐seq study, all of these factors are epidemiologically correlated to COVID‐19 susceptibility and lethality.^36^

Interestingly, A549 cells could be infected by SARS‐CoV‐2, although they do not express ACE2. A recent study has been reported that ENPEP might be another potential receptor for human CoVs demonstrated by single‐cell RNA sequencing.^37^ Therefore, other possible alternative receptors, which could work as an entry receptor to facilitate SARS‐CoV‐2 entry into the host cells, cannot be excluded.

The percentage of ORF1ab and ORF6 in the virus significantly correlated to the expression level of ACE2 in host cells, suggesting that ORF1ab and ORF6 might be synergistically induced in an ACE2‐dependent manner upon SARS‐CoV‐2 infection. It is known that ORF1ab is a subunit of the replicase complex, which is very critical for SARS‐CoV‐2 replication.^38^ ORF6 is a protein that prevents SARS‐CoV‐2‐infected cells from sending signals to the immune system. It also blocks some of the antiviral protein generations of the host cell itself.^39^ These results suggested that ACE2 facilitates SARS‐CoV‐2 infection and replication in host cells, probably through the induction of ORF1ab and ORF6. However, the detailed mechanisms in which ORF1ab and ORF6 are involved in are warranted for further exploration.

Altogether, the present study supplied several lines of evidence that SARS‐CoV‐2 infection triggers immune and interferon responses. Pyrimidine metabolism and steroid hormone biosynthesis also correspond to SARS‐CoV‐2 infection in human lung cells. Also, ACE2 facilitates SARS‐CoV‐2 infection and replication in host cells, probably through the induction of ORF1ab and ORF6. These findings are uniquely in SARS‐CoV‐2 infection, which may help to understand the pathogenesis of COVID‐19 better. The present study highlights the risk and pathogenicity of SARS‐CoV‐2 that we should pay more attention to the threat of COVID‐19.

## CONFLICT OF INTERESTS

The authors declare that there are no conflict of interests.

## AUTHOR CONTRIBUTIONS

Wuping Sun, Shaomin Yang, and Qiwen Deng were responsible for the concept and design of the study. Wuping Sun, Shaomin Yang, Songbin Wu, Zhijian Yu, Jiabin Huang, Xia Zhong, Xiaodong Liu, Lizu Xiao, Hua Zhu, and Qiwen Deng were involved with experimental and analytical aspects of the manuscript. Wuping Sun, Shaomin Yang, and Qiwen Deng performed data interpretation, presentation, and writing of the manuscript. Wuping Sun obtained funding. This manuscript has been read and approved by all authors for publication.

## Supporting information

Supporting information.

## Data Availability

The RNAseq datasets were downloaded from the NCBI GEO database (accession no. GSE147507).
