# Supplementary material for: Transcriptomic analysis reveals novel mechanisms of SARS‐CoV‐2 infection in human lung cells
Source: Immun Inflamm Dis. 2020 Oct 30;8(4):753–62. doi: 10.1002/iid3.366 (PMC7654422; doi:10.1002/iid3.366)
Supplement: Supplementary file 1 — Supporting information. [file IID3-8-753-s001.docx]

**Supplemental materials:**

**Supplemental figure legends**

**Figure S1.** **Gene expression** **data quality of RNA-seq from samples**

**A** Gene expression distribution of each sample. **B** Principal component analysis (PCA) lot for the gene expression level of each sample. **C** Heatmap of the correlation between each sample with pearson method.

**Figure S2. Functional analyses of DEGs by GO classifications**

**A, B, C, and D** The significantly enriched Gene Ontology (GO) terms in molecular function enrichment of A549 cells (A), NHBE cells (B), Calu3 cells (C) and COVID-19 lung biopsy (D) after SARS-CoV-2 infection. **E, F, and G** The significantly enriched Gene Ontology (GO) terms in cellular component enrichment of A549 cells (E), Calu3 cells (F) and COVID-19 lung biopsy (G). The enriched gene number as the abscissa and GO terms is plotted as the ordinate.

**Figure S3. KEGG classifications of DEGs in A549 cells infected with different influenza viruses**

**A, B, and C** The comparison of pathway enrichment in A549 cells infected with respiratory syncytial virus (RSV, A), influenza A virus (IAV, B) and human parainfluenza virus 3 (HPIV3, B) infection. It showed the top 20 significantly enriched Kyoto Encyclopedia of Genes and Genomes (KEGG) pathways. The rich factor as the abscissa and KEGG terms is plotted as the ordinate.

**Figure S4. The expression of human ACE2 in different human lung cells**

**A** The reads count of ACE2 in different types of human lung cells, including A549, NHBE, Calu3 cells, human lung biopsies, and ACE2 over-expressing A549 cells. **B** RT–PCR analysis of the expression of human ACE2 and GAPDH in A549 cells and HEK293T over expressed ACE2 cells. Negative control (NC) lane indicates the result without template.
